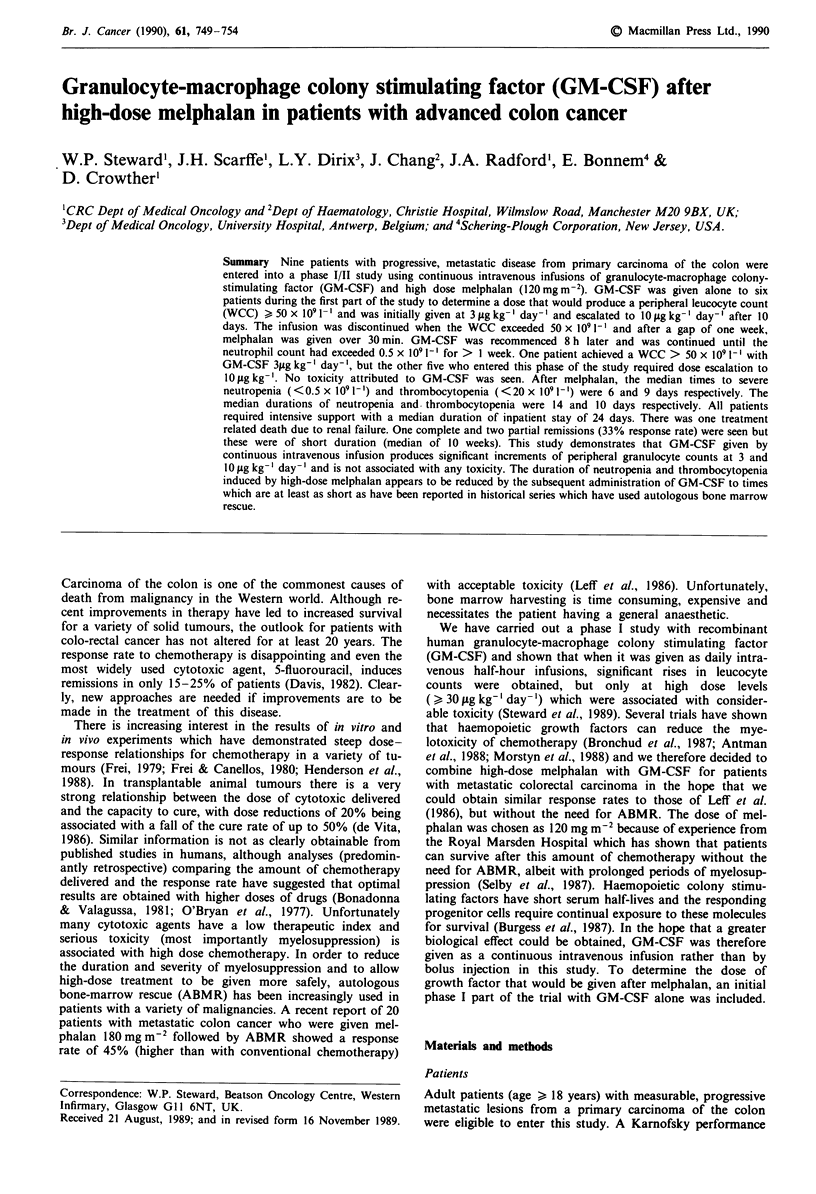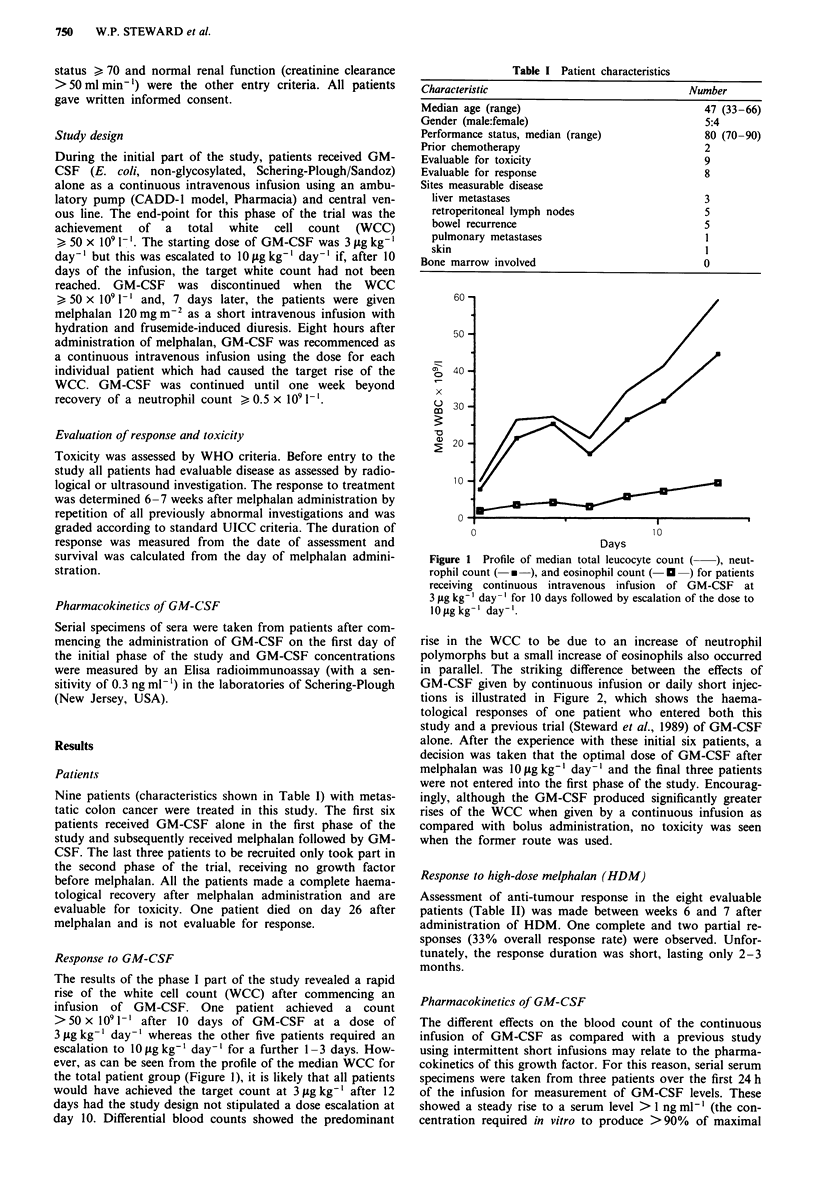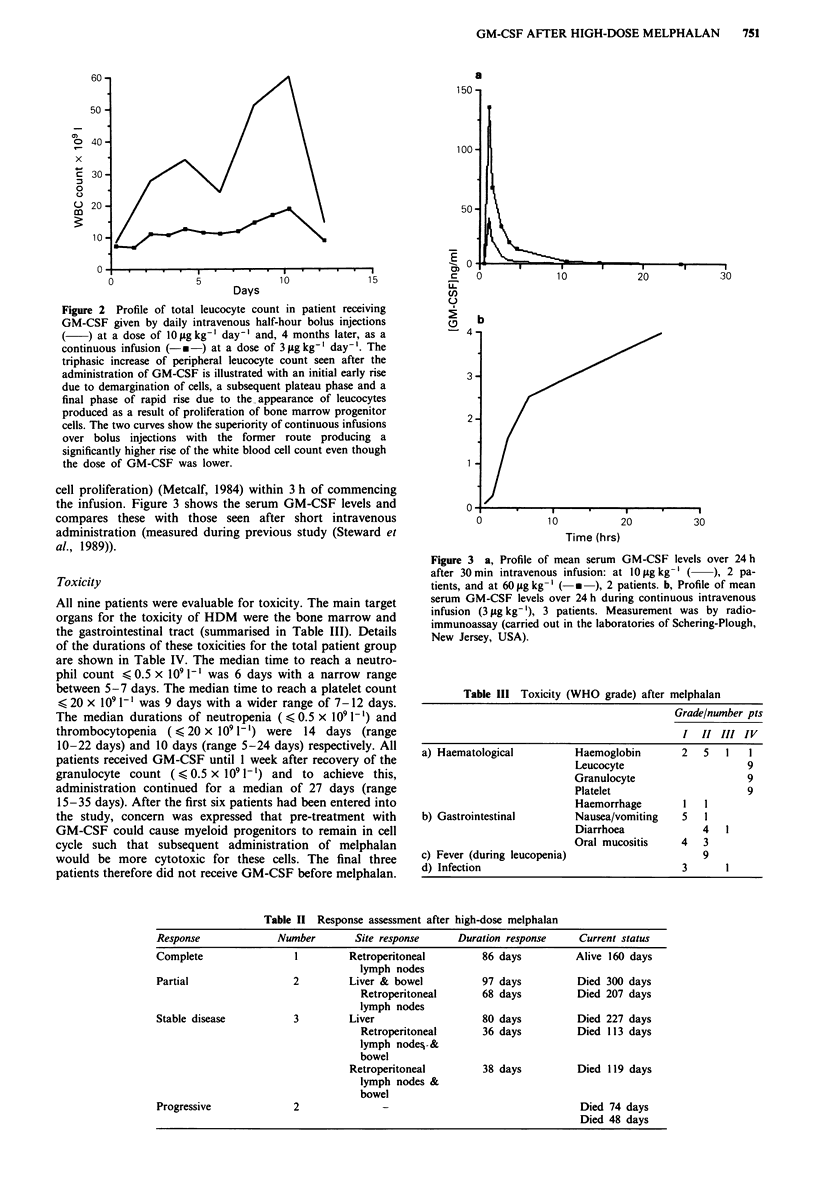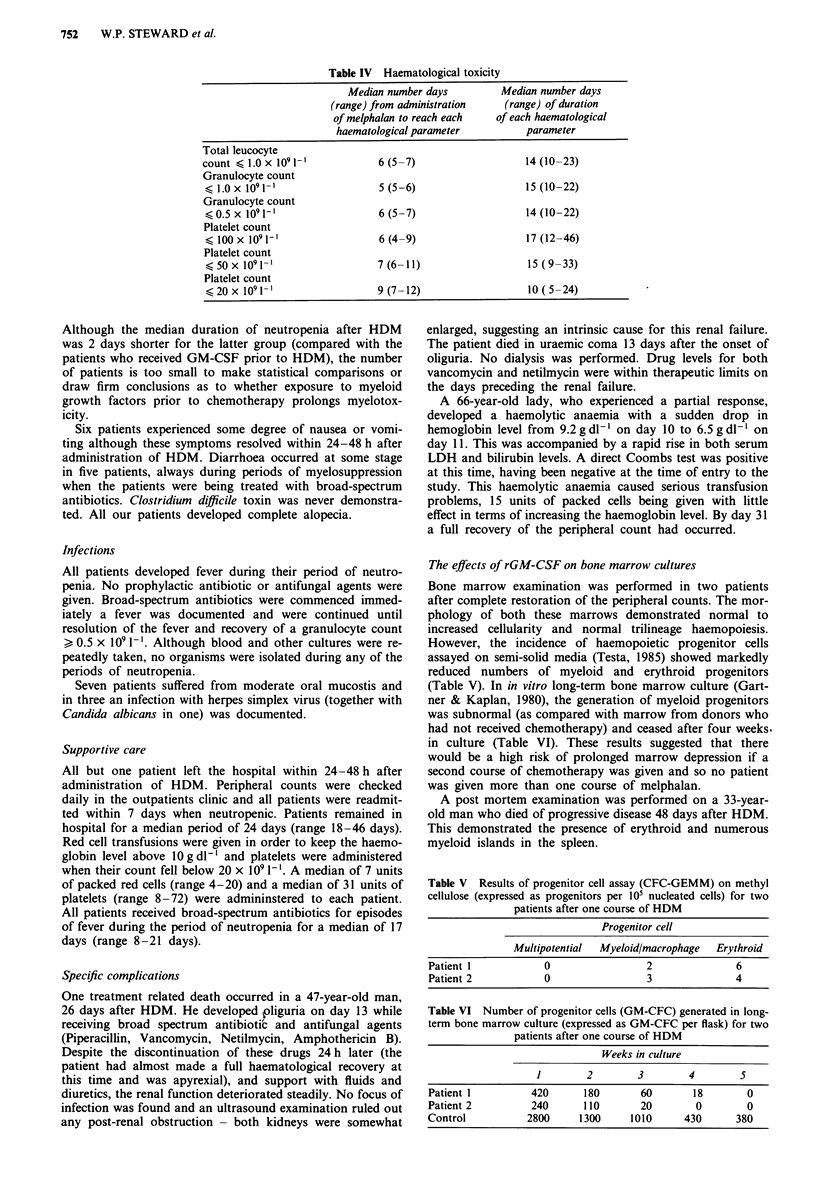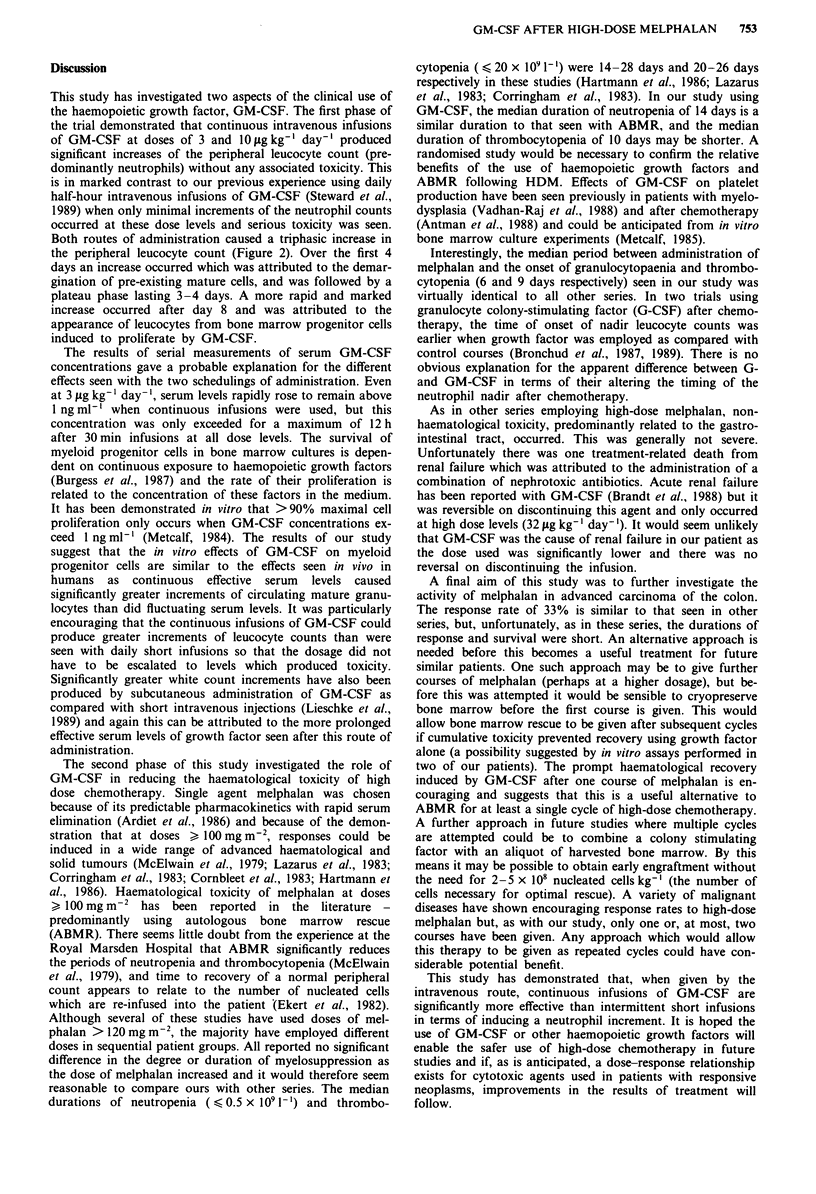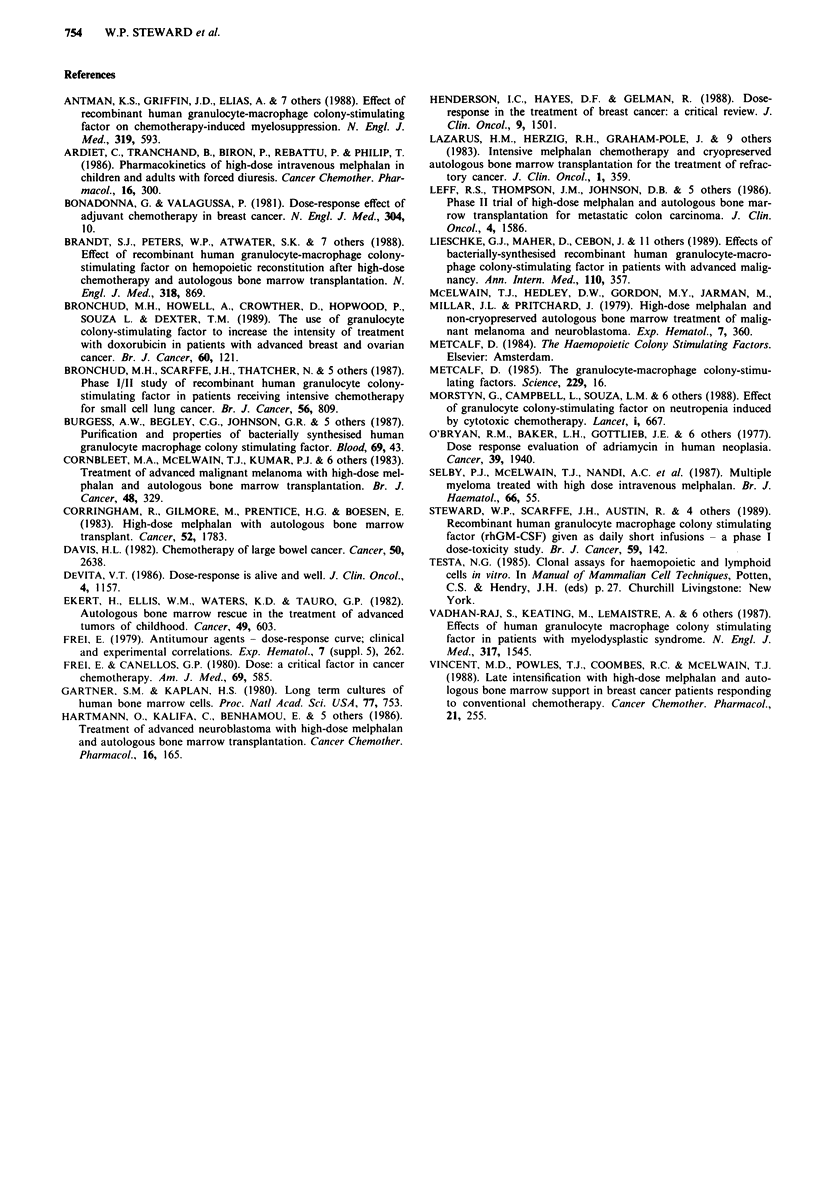# Granulocyte-macrophage colony stimulating factor (GM-CSF) after high-dose melphalan in patients with advanced colon cancer.

**DOI:** 10.1038/bjc.1990.167

**Published:** 1990-05

**Authors:** W. P. Steward, J. H. Scarffe, L. Y. Dirix, J. Chang, J. A. Radford, E. Bonnem, D. Crowther

**Affiliations:** CRC Dept of Medical Oncology, Christie Hospital, Manchester, UK.

## Abstract

Nine patients with progressive, metastatic disease from primary carcinoma of the colon were entered into a phase I/II study using continuous intravenous infusions of granulocyte-macrophage colony-stimulating factor (GM-CSF) and high dose melphalan (120 mg m-2). GM-CSF was given alone to six patients during the first part of the study to determine a dose that would produce a peripheral leucocyte count (WCC) greater than or equal to 50 X 10(9) 1(-1) and was initially given at 3 micrograms kg-1 day-1 and escalated to 10 micrograms kg-1 day-1 after 10 days. The infusion was discontinued when the WCC exceeded 50 X 10(9) 1(-1) and after a gap of one week, melphalan was given over 30 min. GM-CSF was recommenced 8 h later and was continued until the neutrophil count had exceeded 0.5 X 10(9) 1(-1) for greater than 1 week. One patient achieved a WCC greater than 50 X 10(9) 1(-1) with GM-CSF 3 micrograms kg-1 day-1, but the other five who entered this phase of the study required dose escalation to 10 micrograms kg-1. No toxicity attributed to GM-CSF was seen. After melphalan, the median times to severe neutropenia (less than 0.5 X 10(9) 1(-1] and thrombocytopenia (greater than 20 X 10(9) 1(-1] were 6 and 9 days respectively. The median durations of neutropenia and thrombocytopenia were 14 and 10 days respectively. All patients required intensive support with a median duration of inpatient stay of 24 days. There was one treatment related death due to renal failure. One complete and two partial remissions (33% response rate) were seen but these were of short duration (median of 10 weeks). This study demonstrates that GM-CSF given by continuous intravenous infusion produces significant increments of peripheral granulocyte counts at 3 and 10 micrograms kg-1 day-1 and is not associated with any toxicity. The duration of neutropenia and thrombocytopenia induced by high-dose melphalan appears to be reduced by the subsequent administration of GM-CSF to times which are at least as short as have been reported in historical series which have used autologous bone marrow rescue.


*Br. J. Cancer* (1990), **61**, 749–754 © Macmillan Press Ltd., 1990

### Granulocyte-macrophage colony stimulating factor (GM-CSF) after high-dose melphalan in patients with advanced colon cancer

W.P. Steward[1], J.H. Scarffe[1], L.Y. Dirix[3], J. Chang[2], J.A. Radford[1], E. Bonnem[4] & D. Crowther[1]

[1]*CRC Dept of Medical Oncology and* [2]*Dept of Haematology, Christie Hospital, Wilmslow Road, Manchester M20 9BX, UK;* [3]*Dept of Medical Oncology, University Hospital, Antwerp, Belgium; and* [4]*Schering-Plough Corporation, New Jersey, USA.*

**Summary** Nine patients with progressive, metastatic disease from primary carcinoma of the colon were entered into a phase I/II study using continuous intravenous infusions of granulocyte-macrophage colony-stimulating factor (GM-CSF) and high dose melphalan (120 mg m$^{-2}$). GM-CSF was given alone to six patients during the first part of the study to determine a dose that would produce a peripheral leucocyte count (WCC) $\geqslant 50 \times 10^9$ l$^{-1}$ and was initially given at 3 µg kg$^{-1}$ day$^{-1}$ and escalated to 10 µg kg$^{-1}$ day$^{-1}$ after 10 days. The infusion was discontinued when the WCC exceeded $50 \times 10^9$ l$^{-1}$ and after a gap of one week, melphalan was given over 30 min. GM-CSF was recommenced 8 h later and was continued until the neutrophil count had exceeded $0.5 \times 10^9$ l$^{-1}$ for $>$ 1 week. One patient achieved a WCC $> 50 \times 10^9$ l$^{-1}$ with GM-CSF 3µg kg$^{-1}$ day$^{-1}$, but the other five who entered this phase of the study required dose escalation to 10 µg kg$^{-1}$. No toxicity attributed to GM-CSF was seen. After melphalan, the median times to severe neutropenia ($<0.5 \times 10^9$ l$^{-1}$) and thrombocytopenia ($<20 \times 10^9$ l$^{-1}$) were 6 and 9 days respectively. The median durations of neutropenia and thrombocytopenia were 14 and 10 days respectively. All patients required intensive support with a median duration of inpatient stay of 24 days. There was one treatment related death due to renal failure. One complete and two partial remissions (33% response rate) were seen but these were of short duration (median of 10 weeks). This study demonstrates that GM-CSF given by continuous intravenous infusion produces significant increments of peripheral granulocyte counts at 3 and 10 µg kg$^{-1}$ day$^{-1}$ and is not associated with any toxicity. The duration of neutropenia and thrombocytopenia induced by high-dose melphalan appears to be reduced by the subsequent administration of GM-CSF to times which are at least as short as have been reported in historical series which have used autologous bone marrow rescue.

Carcinoma of the colon is one of the commonest causes of death from malignancy in the Western world. Although recent improvements in therapy have led to increased survival for a variety of solid tumours, the outlook for patients with colo-rectal cancer has not altered for at least 20 years. The response rate to chemotherapy is disappointing and even the most widely used cytotoxic agent, 5-fluorouracil, induces remissions in only 15–25% of patients (Davis, 1982). Clearly, new approaches are needed if improvements are to be made in the treatment of this disease.

There is increasing interest in the results of *in vitro* and *in vivo* experiments which have demonstrated steep dose–response relationships for chemotherapy in a variety of tumours (Frei, 1979; Frei & Canellos, 1980; Henderson *et al.*, 1988). In transplantable animal tumours there is a very strong relationship between the dose of cytotoxic delivered and the capacity to cure, with dose reductions of 20% being associated with a fall of the cure rate of up to 50% (de Vita, 1986). Similar information is not as clearly obtainable from published studies in humans, although analyses (predominantly retrospective) comparing the amount of chemotherapy delivered and the response rate have suggested that optimal results are obtained with higher doses of drugs (Bonadonna & Valagussa, 1981; O'Bryan *et al.*, 1977). Unfortunately many cytotoxic agents have a low therapeutic index and serious toxicity (most importantly myelosuppression) is associated with high dose chemotherapy. In order to reduce the duration and severity of myelosuppression and to allow high-dose treatment to be given more safely, autologous bone-marrow rescue (ABMR) has been increasingly used in patients with a variety of malignancies. A recent report of 20 patients with metastatic colon cancer who were given melphalan 180 mg m$^{-2}$ followed by ABMR showed a response rate of 45% (higher than with conventional chemotherapy) with acceptable toxicity (Leff *et al.*, 1986). Unfortunately, bone marrow harvesting is time consuming, expensive and necessitates the patient having a general anaesthetic.

We have carried out a phase I study with recombinant human granulocyte-macrophage colony stimulating factor (GM-CSF) and shown that when it was given as daily intravenous half-hour infusions, significant rises in leucocyte counts were obtained, but only at high dose levels ($\geqslant$ 30 µg kg$^{-1}$ day$^{-1}$) which were associated with considerable toxicity (Steward *et al.*, 1989). Several trials have shown that haemopoietic growth factors can reduce the myelotoxicity of chemotherapy (Bronchud *et al.*, 1987; Antman *et al.*, 1988; Morstyn *et al.*, 1988) and we therefore decided to combine high-dose melphalan with GM-CSF for patients with metastatic colorectal carcinoma in the hope that we could obtain similar response rates to those of Leff *et al.* (1986), but without the need for ABMR. The dose of melphalan was chosen as 120 mg m$^{-2}$ because of experience from the Royal Marsden Hospital which has shown that patients can survive after this amount of chemotherapy without the need for ABMR, albeit with prolonged periods of myelosuppression (Selby *et al.*, 1987). Haemopoietic colony stimulating factors have short serum half-lives and the responding progenitor cells require continual exposure to these molecules for survival (Burgess *et al.*, 1987). In the hope that a greater biological effect could be obtained, GM-CSF was therefore given as a continuous intravenous infusion rather than by bolus injection in this study. To determine the dose of growth factor that would be given after melphalan, an initial phase I part of the trial with GM-CSF alone was included.

### Materials and methods

### *Patients*

Adult patients (age $\geqslant$ 18 years) with measurable, progressive metastatic lesions from a primary carcinoma of the colon were eligible to enter this study. A Karnofsky performance

Correspondence: W.P. Steward, Beatson Oncology Centre, Western Infirmary, Glasgow G11 6NT, UK.
Received 21 August, 1989; and in revised form 16 November 1989.

status $\geqslant 70$ and normal renal function (creatinine clearance $> 50\,ml\,min^{-1}$) were the other entry criteria. All patients gave written informed consent.

*Study design*

During the initial part of the study, patients received GM-CSF (*E. coli*, non-glycosylated, Schering-Plough/Sandoz) alone as a continuous intravenous infusion using an ambulatory pump (CADD-1 model, Pharmacia) and central venous line. The end-point for this phase of the trial was the achievement of a total white cell count (WCC) $\geqslant 50 \times 10^9\,l^{-1}$. The starting dose of GM-CSF was $3\,\mu g\,kg^{-1}\,day^{-1}$ but this was escalated to $10\,\mu g\,kg^{-1}\,day^{-1}$ if, after 10 days of the infusion, the target white count had not been reached. GM-CSF was discontinued when the WCC $\geqslant 50 \times 10^9\,l^{-1}$ and, 7 days later, the patients were given melphalan $120\,mg\,m^{-2}$ as a short intravenous infusion with hydration and frusemide-induced diuresis. Eight hours after administration of melphalan, GM-CSF was recommenced as a continuous intravenous infusion using the dose for each individual patient which had caused the target rise of the WCC. GM-CSF was continued until one week beyond recovery of a neutrophil count $\geqslant 0.5 \times 10^9\,l^{-1}$.

*Evaluation of response and toxicity*

Toxicity was assessed by WHO criteria. Before entry to the study all patients had evaluable disease as assessed by radiological or ultrasound investigation. The response to treatment was determined 6–7 weeks after melphalan administration by repetition of all previously abnormal investigations and was graded according to standard UICC criteria. The duration of response was measured from the date of assessment and survival was calculated from the day of melphalan administration.

*Pharmacokinetics of GM-CSF*

Serial specimens of sera were taken from patients after commencing the administration of GM-CSF on the first day of the initial phase of the study and GM-CSF concentrations were measured by an Elisa radioimmunoassay (with a sensitivity of $0.3\,ng\,ml^{-1}$) in the laboratories of Schering-Plough (New Jersey, USA).

### Results

*Patients*

Nine patients (characteristics shown in Table I) with metastatic colon cancer were treated in this study. The first six patients received GM-CSF alone in the first phase of the study and subsequently received melphalan followed by GM-CSF. The last three patients to be recruited only took part in the second phase of the trial, receiving no growth factor before melphalan. All the patients made a complete haematological recovery after melphalan administration and are evaluable for toxicity. One patient died on day 26 after melphalan and is not evaluable for response.

*Response to GM-CSF*

The results of the phase I part of the study revealed a rapid rise of the white cell count (WCC) after commencing an infusion of GM-CSF. One patient achieved a count $> 50 \times 10^9\,l^{-1}$ after 10 days of GM-CSF at a dose of $3\,\mu g\,kg^{-1}\,day^{-1}$ whereas the other five patients required an escalation to $10\,\mu g\,kg^{-1}\,day^{-1}$ for a further 1–3 days. However, as can be seen from the profile of the median WCC for the total patient group (Figure 1), it is likely that all patients would have achieved the target count at $3\,\mu g\,kg^{-1}$ after 12 days had the study design not stipulated a dose escalation at day 10. Differential blood counts showed the predominant

**Table I** Patient characteristics

| *Characteristic* | *Number* |
|---|---|
| Median age (range) | 47 (33–66) |
| Gender (male:female) | 5:4 |
| Performance status, median (range) | 80 (70–90) |
| Prior chemotherapy | 2 |
| Evaluable for toxicity | 9 |
| Evaluable for response | 8 |
| Sites measurable disease | |
| liver metastases | 3 |
| retroperitoneal lymph nodes | 5 |
| bowel recurrence | 5 |
| pulmonary metastases | 1 |
| skin | 1 |
| Bone marrow involved | 0 |

**Figure 1** Profile of median total leucocyte count (——), neutrophil count (—■—), and eosinophil count (—◘—) for patients receiving continuous intravenous infusion of GM-CSF at $3\,\mu g\,kg^{-1}\,day^{-1}$ for 10 days followed by escalation of the dose to $10\,\mu g\,kg^{-1}\,day^{-1}$.

rise in the WCC to be due to an increase of neutrophil polymorphs but a small increase of eosinophils also occurred in parallel. The striking difference between the effects of GM-CSF given by continuous infusion or daily short injections is illustrated in Figure 2, which shows the haematological responses of one patient who entered both this study and a previous trial (Steward *et al.*, 1989) of GM-CSF alone. After the experience with these initial six patients, a decision was taken that the optimal dose of GM-CSF after melphalan was $10\,\mu g\,kg^{-1}\,day^{-1}$ and the final three patients were not entered into the first phase of the study. Encouragingly, although the GM-CSF produced significantly greater rises of the WCC when given by a continuous infusion as compared with bolus administration, no toxicity was seen when the former route was used.

*Response to high-dose melphalan (HDM)*

Assessment of anti-tumour response in the eight evaluable patients (Table II) was made between weeks 6 and 7 after administration of HDM. One complete and two partial responses (33% overall response rate) were observed. Unfortunately, the response duration was short, lasting only 2–3 months.

*Pharmacokinetics of GM-CSF*

The different effects on the blood count of the continuous infusion of GM-CSF as compared with a previous study using intermittent short infusions may relate to the pharmacokinetics of this growth factor. For this reason, serial serum specimens were taken from three patients over the first 24 h of the infusion for measurement of GM-CSF levels. These showed a steady rise to a serum level $> 1\,ng\,ml^{-1}$ (the concentration required *in vitro* to produce $> 90\%$ of maximal

**Figure 2** Profile of total leucocyte count in patient receiving GM-CSF given by daily intravenous half-hour bolus injections (——) at a dose of $10\,\mu g\,kg^{-1}\,day^{-1}$ and, 4 months later, as a continuous infusion (—■—) at a dose of $3\,\mu g\,kg^{-1}\,day^{-1}$. The triphasic increase of peripheral leucocyte count seen after the administration of GM-CSF is illustrated with an initial early rise due to demargination of cells, a subsequent plateau phase and a final phase of rapid rise due to the appearance of leucocytes produced as a result of proliferation of bone marrow progenitor cells. The two curves show the superiority of continuous infusions over bolus injections with the former route producing a significantly higher rise of the white blood cell count even though the dose of GM-CSF was lower.

cell proliferation) (Metcalf, 1984) within 3 h of commencing the infusion. Figure 3 shows the serum GM-CSF levels and compares these with those seen after short intravenous administration (measured during previous study (Steward *et al.*, 1989)).

*Toxicity*

All nine patients were evaluable for toxicity. The main target organs for the toxicity of HDM were the bone marrow and the gastrointestinal tract (summarised in Table III). Details of the durations of these toxicities for the total patient group are shown in Table IV. The median time to reach a neutrophil count $\leqslant 0.5 \times 10^9\,l^{-1}$ was 6 days with a narrow range between 5–7 days. The median time to reach a platelet count $\leqslant 20 \times 10^9\,l^{-1}$ was 9 days with a wider range of 7–12 days. The median durations of neutropenia ($\leqslant 0.5 \times 10^9\,l^{-1}$) and thrombocytopenia ($\leqslant 20 \times 10^9\,l^{-1}$) were 14 days (range 10–22 days) and 10 days (range 5–24 days) respectively. All patients received GM-CSF until 1 week after recovery of the granulocyte count ($\leqslant 0.5 \times 10^9\,l^{-1}$) and to achieve this, administration continued for a median of 27 days (range 15–35 days). After the first six patients had been entered into the study, concern was expressed that pre-treatment with GM-CSF could cause myeloid progenitors to remain in cell cycle such that subsequent administration of melphalan would be more cytotoxic for these cells. The final three patients therefore did not receive GM-CSF before melphalan.

**Figure 3** **a**, Profile of mean serum GM-CSF levels over 24 h after 30 min intravenous infusion: at $10\,\mu g\,kg^{-1}$ (——), 2 patients, and at $60\,\mu g\,kg^{-1}$ (—■—), 2 patients. **b**, Profile of mean serum GM-CSF levels over 24 h during continuous intravenous infusion ($3\,\mu g\,kg^{-1}$), 3 patients. Measurement was by radioimmunoassay (carried out in the laboratories of Schering-Plough, New Jersey, USA).

**Table III** Toxicity (WHO grade) after melphalan

| | | *Grade/number pts* | | | |
|---|---|---|---|---|---|
| | | *I* | *II* | *III* | *IV* |
| a) Haematological | Haemoglobin | 2 | 5 | 1 | 1 |
| | Leucocyte | | | | 9 |
| | Granulocyte | | | | 9 |
| | Platelet | | | | 9 |
| | Haemorrhage | 1 | 1 | | |
| b) Gastrointestinal | Nausea/vomiting | 5 | 1 | | |
| | Diarrhoea | | 4 | 1 | |
| | Oral mucositis | 4 | 3 | | |
| c) Fever (during leucopenia) | | | 9 | | |
| d) Infection | | 3 | | 1 | |

**Table II** Response assessment after high-dose melphalan

| *Response* | *Number* | *Site response* | *Duration response* | *Current status* |
|---|---|---|---|---|
| Complete | 1 | Retroperitoneal lymph nodes | 86 days | Alive 160 days |
| Partial | 2 | Liver & bowel | 97 days | Died 300 days |
| | | Retroperitoneal lymph nodes | 68 days | Died 207 days |
| Stable disease | 3 | Liver | 80 days | Died 227 days |
| | | Retroperitoneal lymph nodes & bowel | 36 days | Died 113 days |
| | | Retroperitoneal lymph nodes & bowel | 38 days | Died 119 days |
| Progressive | 2 | – | | Died 74 days<br>Died 48 days |

**Table IV** Haematological toxicity

| | *Median number days (range) from administration of melphalan to reach each haematological parameter* | *Median number days (range) of duration of each haematological parameter* |
|---|---|---|
| Total leucocyte count $\leqslant 1.0 \times 10^9 l^{-1}$ | 6 (5–7) | 14 (10–23) |
| Granulocyte count $\leqslant 1.0 \times 10^9 l^{-1}$ | 5 (5–6) | 15 (10–22) |
| Granulocyte count $\leqslant 0.5 \times 10^9 l^{-1}$ | 6 (5–7) | 14 (10–22) |
| Platelet count $\leqslant 100 \times 10^9 l^{-1}$ | 6 (4–9) | 17 (12–46) |
| Platelet count $\leqslant 50 \times 10^9 l^{-1}$ | 7 (6–11) | 15 (9–33) |
| Platelet count $\leqslant 20 \times 10^9 l^{-1}$ | 9 (7–12) | 10 (5–24) |

Although the median duration of neutropenia after HDM was 2 days shorter for the latter group (compared with the patients who received GM-CSF prior to HDM), the number of patients is too small to make statistical comparisons or draw firm conclusions as to whether exposure to myeloid growth factors prior to chemotherapy prolongs myelotoxicity.

Six patients experienced some degree of nausea or vomiting although these symptoms resolved within 24–48 h after administration of HDM. Diarrhoea occurred at some stage in five patients, always during periods of myelosuppression when the patients were being treated with broad-spectrum antibiotics. *Clostridium difficile* toxin was never demonstrated. All our patients developed complete alopecia.

### *Infections*

All patients developed fever during their period of neutropenia. No prophylactic antibiotic or antifungal agents were given. Broad-spectrum antibiotics were commenced immediately a fever was documented and were continued until resolution of the fever and recovery of a granulocyte count $\geqslant 0.5 \times 10^9 l^{-1}$. Although blood and other cultures were repeatedly taken, no organisms were isolated during any of the periods of neutropenia.

Seven patients suffered from moderate oral mucostis and in three an infection with herpes simplex virus (together with *Candida albicans* in one) was documented.

### *Supportive care*

All but one patient left the hospital within 24–48 h after administration of HDM. Peripheral counts were checked daily in the outpatients clinic and all patients were readmitted within 7 days when neutropenic. Patients remained in hospital for a median period of 24 days (range 18–46 days). Red cell transfusions were given in order to keep the haemoglobin level above $10 g dl^{-1}$ and platelets were administered when their count fell below $20 \times 10^9 l^{-1}$. A median of 7 units of packed red cells (range 4–20) and a median of 31 units of platelets (range 8–72) were admininstered to each patient. All patients received broad-spectrum antibiotics for episodes of fever during the period of neutropenia for a median of 17 days (range 8–21 days).

### *Specific complications*

One treatment related death occurred in a 47-year-old man, 26 days after HDM. He developed oliguria on day 13 while receiving broad spectrum antibiotic and antifungal agents (Piperacillin, Vancomycin, Netilmycin, Amphothericin B). Despite the discontinuation of these drugs 24 h later (the patient had almost made a full haematological recovery at this time and was apyrexial), and support with fluids and diuretics, the renal function deteriorated steadily. No focus of infection was found and an ultrasound examination ruled out any post-renal obstruction – both kidneys were somewhat enlarged, suggesting an intrinsic cause for this renal failure. The patient died in uraemic coma 13 days after the onset of oliguria. No dialysis was performed. Drug levels for both vancomycin and netilmycin were within therapeutic limits on the days preceding the renal failure.

A 66-year-old lady, who experienced a partial response, developed a haemolytic anaemia with a sudden drop in hemoglobin level from $9.2 g dl^{-1}$ on day 10 to $6.5 g dl^{-1}$ on day 11. This was accompanied by a rapid rise in both serum LDH and bilirubin levels. A direct Coombs test was positive at this time, having been negative at the time of entry to the study. This haemolytic anaemia caused serious transfusion problems, 15 units of packed cells being given with little effect in terms of increasing the haemoglobin level. By day 31 a full recovery of the peripheral count had occurred.

### *The effects of rGM-CSF on bone marrow cultures*

Bone marrow examination was performed in two patients after complete restoration of the peripheral counts. The morphology of both these marrows demonstrated normal to increased cellularity and normal trilineage haemopoiesis. However, the incidence of haemopoietic progenitor cells assayed on semi-solid media (Testa, 1985) showed markedly reduced numbers of myeloid and erythroid progenitors (Table V). In *in vitro* long-term bone marrow culture (Gartner & Kaplan, 1980), the generation of myeloid progenitors was subnormal (as compared with marrow from donors who had not received chemotherapy) and ceased after four weeks in culture (Table VI). These results suggested that there would be a high risk of prolonged marrow depression if a second course of chemotherapy was given and so no patient was given more than one course of melphalan.

A post mortem examination was performed on a 33-year-old man who died of progressive disease 48 days after HDM. This demonstrated the presence of erythroid and numerous myeloid islands in the spleen.

**Table V** Results of progenitor cell assay (CFC-GEMM) on methyl cellulose (expressed as progenitors per $10^5$ nucleated cells) for two patients after one course of HDM

| | *Progenitor cell* | | |
|---|---|---|---|
| | *Multipotential* | *Myeloid/macrophage* | *Erythroid* |
| Patient 1 | 0 | 2 | 6 |
| Patient 2 | 0 | 3 | 4 |

**Table VI** Number of progenitor cells (GM-CFC) generated in long-term bone marrow culture (expressed as GM-CFC per flask) for two patients after one course of HDM

| | *Weeks in culture* | | | | |
|---|---|---|---|---|---|
| | *1* | *2* | *3* | *4* | *5* |
| Patient 1 | 420 | 180 | 60 | 18 | 0 |
| Patient 2 | 240 | 110 | 20 | 0 | 0 |
| Control | 2800 | 1300 | 1010 | 430 | 380 |

### Discussion

This study has investigated two aspects of the clinical use of the haemopoietic growth factor, GM-CSF. The first phase of the trial demonstrated that continuous intravenous infusions of GM-CSF at doses of 3 and 10 $\mu g\ kg^{-1}\ day^{-1}$ produced significant increases of the peripheral leucocyte count (predominantly neutrophils) without any associated toxicity. This is in marked contrast to our previous experience using daily half-hour intravenous infusions of GM-CSF (Steward *et al.*, 1989) when only minimal increments of the neutrophil counts occurred at these dose levels and serious toxicity was seen. Both routes of administration caused a triphasic increase in the peripheral leucocyte count (Figure 2). Over the first 4 days an increase occurred which was attributed to the demargination of pre-existing mature cells, and was followed by a plateau phase lasting 3–4 days. A more rapid and marked increase occurred after day 8 and was attributed to the appearance of leucocytes from bone marrow progenitor cells induced to proliferate by GM-CSF.

The results of serial measurements of serum GM-CSF concentrations gave a probable explanation for the different effects seen with the two schedulings of administration. Even at 3 $\mu g\ kg^{-1}\ day^{-1}$, serum levels rapidly rose to remain above 1 $ng\ ml^{-1}$ when continuous infusions were used, but this concentration was only exceeded for a maximum of 12 h after 30 min infusions at all dose levels. The survival of myeloid progenitor cells in bone marrow cultures is dependent on continuous exposure to haemopoietic growth factors (Burgess *et al.*, 1987) and the rate of their proliferation is related to the concentration of these factors in the medium. It has been demonstrated *in vitro* that >90% maximal cell proliferation only occurs when GM-CSF concentrations exceed 1 $ng\ ml^{-1}$ (Metcalf, 1984). The results of our study suggest that the *in vitro* effects of GM-CSF on myeloid progenitor cells are similar to the effects seen *in vivo* in humans as continuous effective serum levels caused significantly greater increments of circulating mature granulocytes than did fluctuating serum levels. It was particularly encouraging that the continuous infusions of GM-CSF could produce greater increments of leucocyte counts than were seen with daily short infusions so that the dosage did not have to be escalated to levels which produced toxicity. Significantly greater white count increments have also been produced by subcutaneous administration of GM-CSF as compared with short intravenous injections (Lieschke *et al.*, 1989) and again this can be attributed to the more prolonged effective serum levels of growth factor seen after this route of administration.

The second phase of this study investigated the role of GM-CSF in reducing the haematological toxicity of high dose chemotherapy. Single agent melphalan was chosen because of its predictable pharmacokinetics with rapid serum elimination (Ardiet *et al.*, 1986) and because of the demonstration that at doses $\geqslant 100\ mg\ m^{-2}$, responses could be induced in a wide range of advanced haematological and solid tumours (McElwain *et al.*, 1979; Lazarus *et al.*, 1983; Corringham *et al.*, 1983; Cornbleet *et al.*, 1983; Hartmann *et al.*, 1986). Haematological toxicity of melphalan at doses $\geqslant 100\ mg\ m^{-2}$ has been reported in the literature – predominantly using autologous bone marrow rescue (ABMR). There seems little doubt from the experience at the Royal Marsden Hospital that ABMR significantly reduces the periods of neutropenia and thrombocytopenia (McElwain *et al.*, 1979), and time to recovery of a normal peripheral count appears to relate to the number of nucleated cells which are re-infused into the patient (Ekert *et al.*, 1982). Although several of these studies have used doses of melphalan $>120\ mg\ m^{-2}$, the majority have employed different doses in sequential patient groups. All reported no significant difference in the degree or duration of myelosuppression as the dose of melphalan increased and it would therefore seem reasonable to compare ours with other series. The median durations of neutropenia ($\leqslant 0.5 \times 10^9\ l^{-1}$) and thrombocytopenia ($\leqslant 20 \times 10^9\ l^{-1}$) were 14–28 days and 20–26 days respectively in these studies (Hartmann *et al.*, 1986; Lazarus *et al.*, 1983; Corringham *et al.*, 1983). In our study using GM-CSF, the median duration of neutropenia of 14 days is a similar duration to that seen with ABMR, and the median duration of thrombocytopenia of 10 days may be shorter. A randomised study would be necessary to confirm the relative benefits of the use of haemopoietic growth factors and ABMR following HDM. Effects of GM-CSF on platelet production have been seen previously in patients with myelodysplasia (Vadhan-Raj *et al.*, 1988) and after chemotherapy (Antman *et al.*, 1988) and could be anticipated from *in vitro* bone marrow culture experiments (Metcalf, 1985).

Interestingly, the median period between administration of melphalan and the onset of granulocytopaenia and thrombocytopenia (6 and 9 days respectively) seen in our study was virtually identical to all other series. In two trials using granulocyte colony-stimulating factor (G-CSF) after chemotherapy, the time of onset of nadir leucocyte counts was earlier when growth factor was employed as compared with control courses (Bronchud *et al.*, 1987, 1989). There is no obvious explanation for the apparent difference between G- and GM-CSF in terms of their altering the timing of the neutrophil nadir after chemotherapy.

As in other series employing high-dose melphalan, non-haematological toxicity, predominantly related to the gastro-intestinal tract, occurred. This was generally not severe. Unfortunately there was one treatment-related death from renal failure which was attributed to the administration of a combination of nephrotoxic antibiotics. Acute renal failure has been reported with GM-CSF (Brandt *et al.*, 1988) but it was reversible on discontinuing this agent and only occurred at high dose levels (32 $\mu g\ kg^{-1}\ day^{-1}$). It would seem unlikely that GM-CSF was the cause of renal failure in our patient as the dose used was significantly lower and there was no reversal on discontinuing the infusion.

A final aim of this study was to further investigate the activity of melphalan in advanced carcinoma of the colon. The response rate of 33% is similar to that seen in other series, but, unfortunately, as in these series, the durations of response and survival were short. An alternative approach is needed before this becomes a useful treatment for future similar patients. One such approach may be to give further courses of melphalan (perhaps at a higher dosage), but before this was attempted it would be sensible to cryopreserve bone marrow before the first course is given. This would allow bone marrow rescue to be given after subsequent cycles if cumulative toxicity prevented recovery using growth factor alone (a possibility suggested by *in vitro* assays performed in two of our patients). The prompt haematological recovery induced by GM-CSF after one course of melphalan is encouraging and suggests that this is a useful alternative to ABMR for at least a single cycle of high-dose chemotherapy. A further approach in future studies where multiple cycles are attempted could be to combine a colony stimulating factor with an aliquot of harvested bone marrow. By this means it may be possible to obtain early engraftment without the need for $2-5 \times 10^8$ nucleated cells $kg^{-1}$ (the number of cells necessary for optimal rescue). A variety of malignant diseases have shown encouraging response rates to high-dose melphalan but, as with our study, only one or, at most, two courses have been given. Any approach which would allow this therapy to be given as repeated cycles could have considerable potential benefit.

This study has demonstrated that, when given by the intravenous route, continuous infusions of GM-CSF are significantly more effective than intermittent short infusions in terms of inducing a neutrophil increment. It is hoped the use of GM-CSF or other haemopoietic growth factors will enable the safer use of high-dose chemotherapy in future studies and if, as is anticipated, a dose–response relationship exists for cytotoxic agents used in patients with responsive neoplasms, improvements in the results of treatment will follow.

### References

ANTMAN, K.S., GRIFFIN, J.D., ELIAS, A. & 7 others (1988). Effect of recombinant human granulocyte-macrophage colony-stimulating factor on chemotherapy-induced myelosuppression. *N. Engl. J. Med.*, **319**, 593.

ARDIET, C., TRANCHAND, B., BIRON, P., REBATTU, P. & PHILIP, T. (1986). Pharmacokinetics of high-dose intravenous melphalan in children and adults with forced diuresis. *Cancer Chemother. Pharmacol.*, **16**, 300.

BONADONNA, G. & VALAGUSSA, P. (1981). Dose-response effect of adjuvant chemotherapy in breast cancer. *N. Engl. J. Med.*, **304**, 10.

BRANDT, S.J., PETERS, W.P., ATWATER, S.K. & 7 others (1988). Effect of recombinant human granulocyte-macrophage colony-stimulating factor on hemopoietic reconstitution after high-dose chemotherapy and autologous bone marrow transplantation. *N. Engl. J. Med.*, **318**, 869.

BRONCHUD, M.H., HOWELL, A., CROWTHER, D., HOPWOOD, P., SOUZA L. & DEXTER, T.M. (1989). The use of granulocyte colony-stimulating factor to increase the intensity of treatment with doxorubicin in patients with advanced breast and ovarian cancer. *Br. J. Cancer*, **60**, 121.

BRONCHUD, M.H., SCARFFE, J.H., THATCHER, N. & 5 others (1987). Phase I/II study of recombinant human granulocyte colony-stimulating factor in patients receiving intensive chemotherapy for small cell lung cancer. *Br. J. Cancer*, **56**, 809.

BURGESS, A.W., BEGLEY, C.G., JOHNSON, G.R. & 5 others (1987). Purification and properties of bacterially synthesised human granulocyte macrophage colony stimulating factor. *Blood*, **69**, 43.

CORNBLEET, M.A., MCELWAIN, T.J., KUMAR, P.J. & 6 others (1983). Treatment of advanced malignant melanoma with high-dose melphalan and autologous bone marrow transplantation. *Br. J. Cancer*, **48**, 329.

CORRINGHAM, R., GILMORE, M., PRENTICE, H.G. & BOESEN, E. (1983). High-dose melphalan with autologous bone marrow transplant. *Cancer*, **52**, 1783.

DAVIS, H.L. (1982). Chemotherapy of large bowel cancer. *Cancer*, **50**, 2638.

DEVITA, V.T. (1986). Dose-response is alive and well. *J. Clin. Oncol.*, **4**, 1157.

EKERT, H., ELLIS, W.M., WATERS, K.D. & TAURO, G.P. (1982). Autologous bone marrow rescue in the treatment of advanced tumors of childhood. *Cancer*, **49**, 603.

FREI, E. (1979). Antitumour agents – dose-response curve; clinical and experimental correlations. *Exp. Hematol.*, **7** (suppl. 5), 262.

FREI, E. & CANELLOS, G.P. (1980). Dose: a critical factor in cancer chemotherapy. *Am. J. Med.*, **69**, 585.

GARTNER, S.M. & KAPLAN, H.S. (1980). Long term cultures of human bone marrow cells. *Proc. Natl Acad. Sci. USA*, **77**, 753.

HARTMANN, O., KALIFA, C., BENHAMOU, E. & 5 others (1986). Treatment of advanced neuroblastoma with high-dose melphalan and autologous bone marrow transplantation. *Cancer Chemother. Pharmacol.*, **16**, 165.

HENDERSON, I.C., HAYES, D.F. & GELMAN, R. (1988). Dose-response in the treatment of breast cancer: a critical review. *J. Clin. Oncol.*, **9**, 1501.

LAZARUS, H.M., HERZIG, R.H., GRAHAM-POLE, J. & 9 others (1983). Intensive melphalan chemotherapy and cryopreserved autologous bone marrow transplantation for the treatment of refractory cancer. *J. Clin. Oncol.*, **1**, 359.

LEFF, R.S., THOMPSON, J.M., JOHNSON, D.B. & 5 others (1986). Phase II trial of high-dose melphalan and autologous bone marrow transplantation for metastatic colon carcinoma. *J. Clin. Oncol.*, **4**, 1586.

LIESCHKE, G.J., MAHER, D., CEBON, J. & 11 others (1989). Effects of bacterially-synthesised recombinant human granulocyte-macrophage colony-stimulating factor in patients with advanced malignancy. *Ann. Intern. Med.*, **110**, 357.

MCELWAIN, T.J., HEDLEY, D.W., GORDON, M.Y., JARMAN, M., MILLAR, J.L. & PRITCHARD, J. (1979). High-dose melphalan and non-cryopreserved autologous bone marrow treatment of malignant melanoma and neuroblastoma. *Exp. Hematol.*, **7**, 360.

METCALF, D. (1984). *The Haemopoietic Colony Stimulating Factors*. Elsevier: Amsterdam.

METCALF, D. (1985). The granulocyte-macrophage colony-stimulating factors. *Science*, **229**, 16.

MORSTYN, G., CAMPBELL, L., SOUZA, L.M. & 6 others (1988). Effect of granulocyte colony-stimulating factor on neutropenia induced by cytotoxic chemotherapy. *Lancet*, **i**, 667.

O'BRYAN, R.M., BAKER, L.H., GOTTLIEB, J.E. & 6 others (1977). Dose response evaluation of adriamycin in human neoplasia. *Cancer*, **39**, 1940.

SELBY, P.J., MCELWAIN, T.J., NANDI, A.C. *et al.* (1987). Multiple myeloma treated with high dose intravenous melphalan. *Br. J. Haematol.*, **66**, 55.

STEWARD, W.P., SCARFFE, J.H., AUSTIN, R. & 4 others (1989). Recombinant human granulocyte macrophage colony stimulating factor (rhGM-CSF) given as daily short infusions – a phase I dose-toxicity study. *Br. J. Cancer*, **59**, 142.

TESTA, N.G. (1985). Clonal assays for haemopoietic and lymphoid cells *in vitro*. In *Manual of Mammalian Cell Techniques*, Potten, C.S. & Hendry, J.H. (eds) p. 27. Churchill Livingstone: New York.

VADHAN-RAJ, S., KEATING, M., LEMAISTRE, A. & 6 others (1987). Effects of human granulocyte macrophage colony stimulating factor in patients with myelodysplastic syndrome. *N. Engl. J. Med.*, **317**, 1545.

VINCENT, M.D., POWLES, T.J., COOMBES, R.C. & MCELWAIN, T.J. (1988). Late intensification with high-dose melphalan and autologous bone marrow support in breast cancer patients responding to conventional chemotherapy. *Cancer Chemother. Pharmacol.*, **21**, 255.